# From clinical trials to informing clinical decision-making: a review of patient-reported outcomes in nononcology medicines approved by the European Medicines Agency (2018–2022)

**DOI:** 10.3389/fphar.2025.1536401

**Published:** 2025-04-11

**Authors:** Sarah Sauchelli, Courtney Levy, Ari Gnanasakthy, Vaidehi Dave, Lynda Doward, Kristina A. Fitzgerald, Robyn Carson

**Affiliations:** ^1^ RTI Health Solutions, Manchester, United Kingdom; ^2^ RTI Health Solutions, Durham, NC, United States; ^3^ Patient-Centered Outcomes Research, AbbVie Inc, Florham Park, NJ, United States

**Keywords:** patient-reported outcome (PRO), European Medicines Agency (EMA), summary of product characteristics (SmPC), health-related quality of life (HRQOL), package leaflet, clinical trials, patient-reported outcome measure

## Abstract

**Introduction:**

Information about a medicine published in the Summary of Product Characteristics (SmPC) and the product’s package leaflet by the European Medicines Agency (EMA) is key to communicate its value to prescribers and patients. The aim of this study was to examine the inclusion of statements related to patient-reported outcomes (PROs) in these documents to communicate patients’ perspectives and experiences of new nononcology medicines.

**Methods:**

Nononcology therapeutic indications recommended for approval by the EMA between 2018–2022 were identified. The Public Assessment Report(s) (PAR), SmPC, and package leaflet published for each indication were examined. Information about the indication and characteristics relating to how the PROs were assessed in confirmatory studies was extracted.

**Results:**

Most nononcology therapeutic indications (n = 98/140, 70%) contained PRO trial data but less than 50% (n = 64/140, 46%) had PRO-related statements in the SmPC and/or package leaflet. Most statements described treatment benefit (n = 60/64, 94%). Statements were most likely to be included in the SmPC and/or package leaflet if supported by at least 1 randomized controlled trial (n = 52/71, 73%), the endpoint assessed patient-reported symptoms or symptom burden (n = 56/71, 79%), and/or the PRO(s) were assessed as a primary endpoint (n = 24/24, 100%).

**Discussion:**

Although trial data pertaining to PROs are reviewed when evaluating nononcology drugs, shortfalls persist in the inclusion of PROs when describing treatment benefit in critical documents used to inform treatment decision-making.

## Introduction

The goal of patient-focused drug development is to ensure that the effect of treatment on outcomes which matter most to the target patient population are evaluated and reported to inform regulatory, reimbursement, provider, and patient decision making. Well-constructed measures of patient-reported outcomes (PROs) collect data from the patient’s perspective and form study endpoints to inform on treatment benefit (European Medicines Agency, 2005; Food and Drug Administration, 2009). Collectively, PROs can inform on many aspects of the patient experience of health and treatment, including signs, symptoms, functioning, treatment side effects, overall health status, and health-related quality of life (HRQOL). Specifically, HRQOL refers to the patient’s perspective on their physical, psychological, and social functioning, their ability to carry out daily activities, and their wellbeing (Patrick et al., 2011).

The value of PROs in the drug development process is widely acknowledged (Mercieca-Bebber et al., 2018). PROs as endpoints in trials are critical for evaluating the efficacy of novel treatments for diseases or conditions that require patients to report on their own symptoms, such as migraine and depression (Gnanasakthy et al., 2017). PRO-related endpoints can also complement clinical and laboratory-based endpoints to provide a holistic view of treatment benefit and add context to facilitate the clinical interpretation of trial results (Mercieca-Bebber et al., 2018; Doward et al., 2010). More widely, PRO data collected in clinical trials are also considered valuable by healthcare decision-makers (e.g., regulators, payers, physicians, and patients) to increase knowledge on patients’ experience of disease and treatments, influence policy-making, guide evidence-based practice, and assist in patient advocacy (Cruz Rivera et al., 2020).

Regulatory bodies encourage the incorporation of PROs in clinical trials evaluating clinical efficacy. Since 2006, the Food and Drug Administration (FDA) has published a series of guidance documents for the selection of PROs to evaluate in clinical trials, and the development of PRO measures that are clinically relevant to patients and appropriate for use in confirmatory clinical trials (Food and Drug Administration, 2022a; Food and Drug Administration, 2022b; Food and Drug Administration, 2023). Similarly, the European Medicines Agency (EMA) has been outlining the value of PRO data from the regulatory perspective (e.g., EMA’s reflection paper on oncology drug development (European Medicines Agency, 2014)), and in 2020 the EMA launched a new strategy to incorporate patient experience data in the risk-benefit evaluation of medicines (European Medicines Agency, 2020; European Medicines Agency, 2022b).

PRO-related endpoints can be used to support product value messaging in the European Union (EU) and select countries in the European Economic Area (EEA). Drug manufacturers can use PRO data to substantiate inclusion of language in documents published by the EMA that communicate the value of treatment to healthcare providers, payers, and patients. For example, the product information documents published by the EMA after a product is approved and includes the product’s package leaflet and the Summary of Product Characteristics (SmPC). The SmPC is made readily available to healthcare professionals and other prescribers via multiple avenues (e.g., national medicine databases, electronic prescribing systems) to inform on the use of a medicinal product. Statements in the SmPC and package leaflet that convey the patient-reported product value (e.g., improvements in symptoms or HRQOL) can contribute to treatment decision-making in clinical practice. This approach for communicating patient-reported treatment benefit differs from the FDA product labelling, which enables direct-to-consumer advertising.

The contents of the SmPC and package leaflet are contingent on the EMA’s scientific review of the quality of the evidence submitted by the manufacturer in their marketing authorization application (MAA). Following their review of the MAA, the EMA’s scientific review committee issues a Public Assessment Report (PAR); a comprehensive document detailing the evaluation of a medicine and reasoning behind the EMA’s decision on whether to recommend the medicine’s approval or rejection for marketing authorization. The PAR serves as reference document to ensure the contents of the SmPC accurately reflect the scientific assessment conducted by EMA reviewers and align with regulatory requirements for the safe and effective use of medicinal products.

The PAR, the SmPC and the package leaflet are published by the EMA on its website as part of the European Public Assessment Reports (EPARs), shortly after a decision is reached regarding marketing authorization of the medicinal product (see the EMA website (European Medicines Agency, 2010) and Supplementary Material 1 for further details). While the PAR will present EMA reviewers’ evaluation of the data presented by the manufacturer, the SmPC and package leaflet will only include statements on treatment effect that have been approved by the EMA for the purposes of giving information to prescribers and patients.

PRO-related statements on treatment effect accepted by the EMA for inclusion in the SmPC and package leaflet elevate and characterize patients’ perspectives and experiences of a treatment for a wide range of stakeholders. The aim of this study was to gain insight into the approval of PRO-related statements to communicate product value of nononcology medicines marketed in the EU and select countries in the EEA between 2018 and 2022. Specifically, the objectives were to: (a) assess the frequency with which PRO data reviewed by the EMA’s scientific review committee (as presented in the PAR) result in PRO-related statements of treatment effect in the SmPC and package leaflet, and (b) explore patterns in the way PROs are incorporated in clinical trials that could contribute to the approval of PRO-related statements in these documents.

## Methods

Annual Summary documents published by the EMA were reviewed to identify all medicines recommended for approval between 1 January 2018 and 31 December 2022 (inclusive). The following medicines were excluded from data collection: medicines indicated to treat malignant neoplasms; medicines indicated for use as diagnostic tools; medicines that did not contain new active substances; and COVID-19 vaccines. The Summary of Product Positive Opinion documents were then examined for each medicine to identify all therapeutic indications approved for the medicine since its initial marketing authorization and up to 31 December 2022.

The EPAR documents of each therapeutic indication were reviewed, including the medicine overview, all published PARs for the indication, the SmPC, and the package leaflet (Supplementary Material 1). Key characteristics (e.g., brand and generic name, market authorization holder, target population) were extracted for each indication. The therapeutic specialty of each indication was identified using *the International Classification of Diseases, 10th Revision* (ICD-10). Therapeutic specialties were then classified into PRO-dependent or non–PRO-dependent, in line with previous research (Gnanasakthy et al., 2017). PRO-dependent therapeutic specialties encompass therapeutic indications for diseases in which clinical efficacy has been traditionally assessed via PRO-related endpoints (e.g., diseases of the nervous system). Non–PRO-dependent specialties comprise indications traditionally supported by other outcomes, such as biomarker, laboratory, or other types of clinical outcome data (e.g., endocrine, nutritional, and metabolic diseases) (Gnanasakthy et al., 2017).

Characteristics of the confirmatory studies for each indication (e.g., study design, baseline sample size) were also collected. Confirmatory studies were defined as any clinical efficacy study referred to as “pivotal” or “main” in the PAR that provide the critical evidence for the EMA’s recommendation to approve the medicine. Indications for which clinical efficacy was inferred from immunogenicity trials, human challenge trials, pharmacokinetic studies, or animal studies were excluded.

All references to PROs in confirmatory studies as reported in the PAR were examined. Any available information on PRO measures used, the PRO concepts assessed, endpoint placement of a PRO, and any statistical and/or clinical significance reported was extracted. PROs were classified based on the language reported in the EPAR documents, and according to the following concept categories: symptoms and symptom burden, functional status, HRQOL, health status, patient experience of care, and other (Cleeland, 2007; Cella et al., 2015; Gnanasakthy et al., 2021). Where the term “quality of life” was used in the documents examined, it was assumed that the target concept was HRQOL. PRO measures were classified according to the following categories: disease-specific, generic (i.e., instruments designed for use in multiple disease areas), trial-specific (i.e., instruments that are not published and standardized, but have been developed bespoke for a clinical trial program), and composite measures (e.g., measures that include both PROs and other clinical outcome data). These categories were not mutually exclusive.

Finally, PRO-related statements of treatment effect were extracted from the SmPC and package leaflet. The statements were grouped into the following categories, based on the language in the SmPC or package leaflet, or supporting PRO data described in the PAR: statements of treatment benefit, when information presented described a positive treatment effect on the PRO; noninferiority of treatment, when PRO data were presented to communicate noninferiority of treatment; no treatment benefit, when information presented indicated that treatment did not have the predicted effect on a PRO; or other, when the intended message of a PRO-related statement in the SmPC or package leaflet could not be interpreted.

## Results

A total of 205 new active substances were reviewed by the EMA and approved between 2018 and 2022, of which 136 had nononcology therapeutic indications that met the criteria for inclusion in this review. Eight substances were approved for more than 1 nononcology indication during the 5-year period. Therefore, a total of 140 therapeutic indications were identified for review (Supplementary Material 2).

Most therapeutic indications examined (n = 98/140, 70%) were supported by PRO data in confirmatory studies, as reported in the PAR. Almost half of therapeutic indications included PRO-related statements in the SmPC and/or package leaflet (n = 64/140, 46%); of which 1 indication (Myalepta for the treatment of complications of leptin deficiency in patients with lipodystrophy (European Medicines Agency, 2018a) contained a PRO-related statement in the package leaflet, with no supporting PRO data available. The remaining 63 indications with PRO-related statements were supported by PRO data presented in the PAR.

When considering only indications with PRO data reported in the PAR (Table 1), most indications (n = 63/98, 64%) had PRO-related statements approved for inclusion in the SmPC and/or package leaflet. Over a third of indications were supported by PRO data in confirmatory studies, but statements on the effect of treatment on the PROs were not in the SmPC and package leaflet. Therefore, inclusion of PRO data in confirmatory studies does not always result in PRO-related statements in the SmPC and/or package leaflet.

**TABLE 1:** Proportion of indications recommended for approval by the EMA that are supported by PRO data and have PRO-related statements in the SmPC and package leaflet, across therapeutic specialties (N = 140).

Therapeutic specialty (ICD-10)	Indications approved, N	Indications with PRO data, n (% of indications)	Indications with PRO-related statements of treatment effect, n (% of indications with PRO data^a^)
**PRO-dependent**	**73**	**57 (78.1)**	**41 (71.9)**
Diseases of the blood and blood-forming organs and certain disorders involving the immune mechanism	18	14 (77.8)	5 (35.7)
Diseases of the nervous system	17	14 (82.4)	13 (92.9)
Diseases of the skin and subcutaneous tissue	10	9 (90.0)	8 (88.9)
Diseases of the musculoskeletal system and connective tissue	9	7 (77.8)	3 (42.9)
Diseases of the respiratory system	6	4 (66.7)	4 (100.0)
Diseases of the eye and adnexa	5	3 (60.0)	2 (66.7)
Diseases of the digestive system	4	4 (100.0)	4 (100.0)
Diseases of the genitourinary system	3	2 (66.7)	2 (100.0)
Mental, behavioral, and neurodevelopmental disorders	1	0 (0.0)	0 (0.0)
**Non–PRO-dependent**	**67**	**41 (61.2)**	**22 (53.7)**
Endocrine, nutritional, and metabolic diseases	29	20 (69.0)	14 (70.0)
Certain infectious and parasitic diseases	13	4 (30.8)	1 (25.0)
Factors influencing health status and contact with health services	8	3 (37.5)	2 (66.7)
Codes for special purposes	7	7 (100.0)	2 (28.6)
Neoplasms	3	3 (100.0)	2 (66.7)
Diseases of the circulatory system	3	1 (33.3)	1 (100.0)
Congenital malformations, deformations, and chromosomal abnormalities	2	2 (100.0)	0 (0.0)
Symptoms, signs, and abnormal clinical and laboratory findings, not elsewhere classified	2	1 (50.0)	0 (0.0)
**Total therapeutic indications**	**140**	**98 (70.0)**	**63 (64.3)**

EMA, European Medicines Agency; ICD-10, *International Classification of Diseases, 10th Revision*; PAR, Public Assessment Report; PRO, patient-reported outcome; SmPC, Summary of Product Characteristics.

^a^ Percentage is derived from dividing the number of indications with PRO-related statements in SmPC, and/or package leaflet by the number of indications with PRO, data in the PAR.

Note: For 1 indication (European Medicines Agency, 2018a), PRO language was identified in the package leaflet that could only be assessed via PRO(s) (i.e., hunger and energy levels), but no information was found on the PROs in the corresponding SmPC or PAR. Data relating to this indication are excluded from this table.

As shown in Table 1, therapeutic specialties classified as PRO-dependent were more likely to comprise indications supported by PRO data (n = 57/73, 78%) compared with non-PRO-dependent specialties (n = 41/67, 61%). Similarly, PRO-related statements were also more frequent for indications pertaining to PRO-dependent specialties (n = 41/57, 72% of indications supported by PRO data) compared with indications pertaining to non-PRO-dependent specialties (n = 22/41, 54% of indications supported by PRO data). However, whether or not an indication was PRO-dependent did not determine the likelihood that PRO-related statements were included in the SmPC and/or package leaflet. For example, the proportion of indications for endocrine, nutritional, and metabolic diseases (classified as non-PRO-dependent) with PRO-related statements in these key documents (n = 14/20, 70% of indications with PRO data) was higher than that for indications for diseases of the blood and blood-forming organs (PRO-dependent; n = 5/14, 36%).

### Study design

Most indications with PRO data (n = 71/98, 72%) were supported by evidence derived from randomized controlled trials (RCTs). As shown in Table 2, PRO-related statements were also most likely to be included in the SmPC and/or package leaflet if confirmatory studies had an RCT design (n = 52/71, 73%). Indications supported by confirmatory evidence from single-arm trials were the least likely to include PRO-related statements in these documents (n = 1/9, 11%).

**TABLE 2:** Inclusion of PRO-related statements among indications with PRO data, across study types (N = 98).

Design of confirmatory studies	Indications with PRO data, n	Indications with PRO-related statements of treatment effect, n (% of indications with PRO data^a^)
Randomized controlled trial	71	52 (73.2)
Multiple trials with different designs	10	6 (60.0)
Randomized open-label trial	6	3 (50.0)
Single-arm trial^b^	9	1 (11.1)
Mixed design^ c^	1	1 (100.0)
Partially randomized, open-label trial	1	0 (0.0)
Total therapeutic indications	98	63 (64.3)

PAR, Public Assessment Report; PRO, patient-reported outcome; SmPC, Summary of Product Characteristics.

Notes: “Multiple trials with mixed designs” is defined as more than 1 trial with different study designs, including randomized controlled trial, randomized open-label trial, and single-arm trial.

^a^ Percentage is derived from dividing the number of indications with PRO-related statements in SmPC and/or package leaflet by the number of indications with PRO data in the PAR.

^b^ For 1 indication (European Medicines Agency, 2018a) supported by confirmatory evidence from an open-label, single-arm trial, PRO language was identified in the package leaflet that could only be assessed via PRO(s) (i.e., hunger and energy levels), but no information was found on PROs in the corresponding PAR. Data relating to this indication are excluded from this table.

^c^ Study design comprises open-label eligibility period, a randomized controlled discontinuation trial period, and a long-term open-label extension period.

### Measures

Most indications with PRO data (n = 84/98, 86%) used at least 1 disease-specific PRO measure in confirmatory studies (Table 3). The most widely used disease-specific PRO measures were patient-reported symptom diaries (n = 24), followed by the Dermatology Quality of Life Index (n = 8), and the Hemophilia Quality of Life Questionnaire for Adults (n = 5). Many therapeutic indications (n = 62/98) also reported the use of generic PRO measures (Table 3); the most frequently used measures were any version of the EQ-5D (n = 35), any version of the SF-36 Health Survey (n = 21), and the Functional Assessment of Chronic Illness Therapy-Fatigue scale (n = 13).

**TABLE 3:** Types of PROMs used across indications with PRO data (N = 98).

Type of PROM	Therapeutic indications with PRO data, n (%)
Disease-specific	84 (85.7)
Generic	62 (63.3)
Trial-specific	45 (45.9)
Composite	10 (10.2)
PROM(s) not reported	4 (4.1)
**Total therapeutic indications**	**98**

EMA, European Medicines Agency; PAR, Public Assessment Report; PRO, patient-reported outcome; PROM, patient-reported outcome measure; SmPC, Summary of Product Characteristics.

Note: For 1 indication (European Medicines Agency, 2018a), PRO language was identified in the package leaflet that could only be assessed via PRO(s) (i.e., hunger and energy levels), but no information was found on the PROs in the corresponding SmPC or PAR. Data relating to this indication are excluded from this table.

Variations of the EQ-5D used included the EQ-5D-5L index (n = 23), EQ-5D visual analog scale (VAS) (n = 20), EQ-5D-3L index (n = 5), an unspecified version of the EQ-5D (n = 5), and EQ-5D-Y (n = 1). The EQ-5D was described as a measure of HRQOL in the PAR of 26 indications, regardless of whether results presented were based on the index or VAS scores.

### Endpoints

Most indications with supporting PRO data in confirmatory studies included PRO-related endpoints used to assess symptoms or symptom burden (n = 71/98, 72%), followed by HRQOL (n = 61/98, 62%), functional status (n = 35/98, 36%), health status (n = 8/98, 8%), and patient experience of treatment such as treatment burden (n = 7/98, 7%) (Table 4). Endpoints were categorized according to the type of PRO measured (e.g., symptom or HRQOL) and based on their role in the trial (i.e., a primary endpoint that is the main outcome used to determine if a treatment is effective and is statistically powered to detect an effect, or a nonprimary endpoint that may or may not be statistically powered to detect an effect and is normally used to provide supporting evidence of treatment efficacy).

**TABLE 4:** Inclusion of PRO-related statements among indications with PRO data, across outcomes and endpoint characteristics (N = 98).

Category	Indications with PRO data, n	Indications with PRO-related statements of treatment effect, n (% of indications with PRO data^a^)
Placement of PRO-related endpoints		
Primary	24	24 (100.0)
Nonprimary^b^	98	57 (58.2)^c^
Type of concept assessed		
Symptoms or symptom burden	71	56 (78.9)
HRQOL	61	23 (37.7)
Functional status	35	19 (54.3)
Health status^ d^	8	3 (37.5)
Patient experience of care	7	0 (0.0)
Total therapeutic indications	98	63 (64.3)

HRQOL, health-related quality of life; PAR, Public Assessment Report; PRO, patient-reported outcome; SmPC, Summary of Product Characteristics; VAS, visual analog scale.

Note: For 1 indication (European Medicines Agency, 2018a), PRO language was identified in the package leaflet that could only be assessed via PRO(s) (i.e., hunger and energy levels), but no information was found on the PROs in the corresponding SmPC or PAR.

Data relating to this indication are excluded from this table.

^a^ Percentage is derived from dividing the number of indications with PRO-related statements in SmPC and/or package leaflet by the number of indications with PRO data in the PAR.

^b^ The nonprimary endpoint category includes multiplicity-adjusted endpoints.

^c^ For 2 indications (European Medicines Agency, 2021a; European Medicines Agency, 2022a), PROs were assessed only as nonprimary endpoints, but it was not possible to determine if the endpoints supported the PRO-related value messages in the SmPC and package leaflet.

^d^ Includes only indications whereby the PAR stated assessment of health status. Indications supported by the use of EQ-5D VAS have not been included unless the PAR stated the EQ-5D VAS was used to assess health status.

PRO-related endpoints described in the PAR did not always result in the inclusion of PRO-related statements in the SmPC or package leaflet. Nonetheless, PRO-related statements in these documents were most likely when the endpoint was used to assess symptom and symptom burden (n = 56/71, 79%). Although there were more indications with PRO data to support treatment effect on HRQOL than functional status, inclusion of HRQOL-related endpoints was less likely to result in the relevant information being included in the SmPC and/or package leaflet, compared with endpoints related to functional status (n = 23/61, 38% and n = 19/35, 54%, respectively).

All indications with PRO data were supported by PROs assessed as a nonprimary endpoint (n = 98/98, 100%), but just over half (n = 57/98, 58%) had statements on treatment effect in the SmPC and/or package leaflet derived from these nonprimary endpoint(s). In contrast, all 24 indications with a PRO assessed as a trial primary endpoint resulted in the corresponding trial results being reported in the SmPC and/or package leaflet (n = 24/24, 100%) (Table 4). The primary endpoint is analyzed first in the testing hierarchy and must be statistically significant for analysis of nonprimary endpoints to continue.

Table 5 presents placement of PRO-related endpoints across therapeutic specialties. As expected, over a third of indications from PRO-dependent therapeutic specialties (n = 22/57, 39%) were supported by confirmatory studies with a primary endpoint based on a PRO, whereas this was the case for only 2 of the 41 (5%) indications from non-PRO-dependent therapeutic specialties.

**TABLE 5:** Types of PRO endpoints documented for indications with PRO data, by therapeutic specialty (N = 98).

Specialty/disease	Therapeutic indications with PRO data, N	Primary endpoints, n (%)	Nonprimary endpoints, n (%)
**PRO-dependent**	**57**	**22 (38.6)**	**57 (100.0)**
Diseases of the nervous system	14	9 (64.3)	14 (100.0)
Diseases of the blood and blood-forming organs and certain disorders involving the immune mechanism	14	0 (0.0)	14 (100.0)
Diseases of the skin and subcutaneous tissue	9	2 (22.2)	9 (100.0)
Diseases of the musculoskeletal system and connective tissue	7	2 (28.6)	7 (100.0)
Diseases of the digestive system	4	4 (100.0)	4 (100.0)
Diseases of the respiratory system	4	4 (100.0)	4 (100.0)
Diseases of the eye and adnexa	3	0 (0.0)	3 (100.0)
Diseases of the genitourinary system	2	1 (50.0)	2 (100.0)
**Non–PRO-dependent**	**41**	**2 (4.9)**	**41 (100.0)**
Endocrine, nutritional, and metabolic diseases	20	2 (10.0)	20 (100.0)
Codes for special purposes	7	0 (0.0)	7 (100.0)
Certain infectious and parasitic diseases	4	0 (0.0)	4 (100.0)
Neoplasms	3	0 (0.0)	3 (100.0)
Factors influencing health status and contact with health services	3	0 (0.0)	3 (100.0)
Congenital malformations, deformations, and chromosomal abnormalities	2	0 (0.0)	2 (100.0)
Diseases of the circulatory system	1	0 (0.0)	1 (100.0)
Symptoms, signs, and abnormal clinical and laboratory findings, not elsewhere classified	1	0 (0.0)	1 (100.0)
**Total therapeutic indications**	**98**	**24 (24.5)**	**98 (100.0)**

PAR, Public Assessment Report; PRO, patient-reported outcome; SmPC, Summary of Product Characteristics.

Note: For 1 indication (European Medicines Agency, 2018a), PRO language was identified in the package leaflet that could only be assessed via PRO(s) (i.e., hunger and energy levels), but no information was found on the PROs in the corresponding SmPC or PAR. Data relating to this indication are excluded from this table.

### Type of PRO-related statement

Table 6 presents the statements on the effect of treatment on PROs included in the SmPC and/or package leaflet of all indications examined. Almost all PRO-related statements communicated the benefit of treatment on at least 1 PRO (n = 60/64, 94%). A smaller proportion of statements described noninferiority of treatment (3/64, 5%) or reported a null treatment effect on a given PRO (n = 2/64, 3%). For five indications, the SmPC included both a statement on the noninferiority of treatment on a PRO, as well as a statement on the superiority of treatment on another PRO.

**TABLE 6:** Types of PRO-related statement in the SmPC and package leaflet across indications (N = 64).

Statement of effect of treatment on PRO	N (%)	Example (brand name/generic name; disease, MAH, year of approval)
Treatment benefit	60 (93.8)	Patients treated with erenumab had a **clinically relevant and statistically significant reduction from baseline in the frequency of migraine days from Months 4 to 6** (Figure 2) compared to patients receiving placebo. Differences from placebo were observed from Month 1 onwards. (European Medicines Agency 2018b)
Noninferiority of treatment	3 (4.7)	Symptoms of inattention and mood were also evaluated during this period. **No differences^ a^ were observed in inattention and mood** between patients randomised to placebo versus those randomised to Palynziq during this 8-week duration. (European Medicines Agency, 2019)
No treatment benefit	2 (3.1)	**Change from baseline to week 24 in pain or fatigue were not met** in studies BN40898 and BN40900. (European Medicines Agency, 2021b)
Other^ b^	4 (6.3)	Natural leptin is produced by fatty tissue and has many functions in the body including: - controlling how hungry you feel and your energy levels - helping the insulin in your body manage sugar levels. Metreleptin works by copying the effects of leptin. This improves the ability of the body to control energy levels. (European Medicines Agency, 2018a)

MAH, market authorization holder; NMOSD, neuromyelitis optica spectrum disorder; PAR, Public Assessment Report; PRO, patient-reported outcome; SmPC, Summary of Product Characteristics.

Notes: Indications with multiple PRO endpoints can contain value messages. Statements of treatment effect included have been derived from primary and/or nonprimary PRO endpoints.

^a^ Study described is a randomized discontinuation trial.

^b^ When the intended message in the SmPC and package leaflet could not be classified.

Note: For 1 indication (European Medicines Agency, 2018a), PRO language was identified in the package leaflet that could only be assessed via PRO(s) (i.e., hunger and energy levels), but no information was found on the PROs in the corresponding SmPC or PAR.

Data relating to this indication have been included in this table.

## Discussion

The present study examined 140 nononcology therapeutic indications reviewed by the EMA between 2018 and 2022. Most indications identified (n = 98/140, 70%) were supported by the inclusion of PRO data in confirmatory studies. However, just under half of nononcology indications examined in this review (n = 64/140, 46%) had at least 1 PRO-related statement on treatment effect in the SmPC and/or package leaflet. Most PRO-related statements in these documents described the benefit of treatment from the patient perspective. In-depth review of the PRO data demonstrated that the approval of PRO-related statements was most likely if the supporting evidence was derived from at least 1 RCT, if the endpoint assessed PROs related to symptoms or symptom burden, and if the PRO was included in the trial to construct a primary endpoint. Notably, symptom PROs tend to be placed higher in the testing hierarchy than other types of endpoints, often because they are more proximal concepts, which could contribute to their more frequent inclusion in the SmPC and package leaflet.

Previous studies have shown a rise in PRO-related endpoints in clinical trials (Scoggins and Patrick, 2009; Vodicka et al., 2015). A review of products recommended for approval by the EMA between 2008 and 2012 found PRO data were included in the efficacy evidence for 46% of the 180 products (Bansal et al., 2015). In contrast, this review showed that, between 2018 and 2022, PRO data were used to support 70% of therapeutic indications recommended for approval. Although a like-for-like comparison between reviews is not possible, findings suggest that the patient-reported experience of disease is increasingly valued in the evaluation of treatment efficacy.

Inclusion of PROs in confirmatory studies, however, does not yet guarantee EMA approval of PRO-related statements in the SmPC and/or package leaflet; in this review, a third of indications did not have PRO-related statements in these documents despite the presence of PRO data in the evidence of treatment efficacy. An even lower success rate has been observed for oncology medicines, where PRO endpoints are often not multiplicity adjusted; reviews of oncology indications recommended for approval by the EMA have found PRO-related statements in only 20%–30% of the SmPCs of indications identified (Gnanasakthy et al., 2023; Cella et al., 2022). In their review of EPAR documents of indications for oncology medicinal products approved between 2017 and 2020, Teixera and colleagues (Teixeira et al., 2022) found that 100 out of 128 (78%) oncology indications approved included PRO measures in the confirmatory studies, 76 (60%) had EMA reviewer comments on PROs, but only 22 (17%) were approved to include PRO-related statements in the SmPC. The authors found critique from EMA reviewers on the methods used to assess PROs and the reporting of PRO data in the evidence submitted, which might have affected the inclusion of the corresponding information on treatment effect in the SmPC (Teixeira et al., 2022).

There are multiple challenges in the implementation and reporting of PROs that can contribute to their exclusion from documents used to communicate product value (Mercieca-Bebber et al., 2018). In rare diseases, for example, the target population for new medicines may be too small to conduct RCTs (European Medicines Agency, 2023). PROs assessed in studies using a single-arm design are vulnerable to responder bias due to the absence of a control group (Liu et al., 2023). Shortfalls in the quality of PRO data presented in the MAA could contribute to the EMA reviewers advising against their inclusion in the SmPC and/or package leaflet (Cella et al., 2022).

It is noteworthy that development of endpoint testing strategies often involve multiple endpoints, including PRO-related endpoints, to evaluate treatment efficacy and safety of a new product. Not all endpoints can be included in the statistical hierarchy, and manufacturers must often make tradeoffs when determining which endpoints to elevate in the statistical hierarchy and the placement of endpoints in the hierarchy to align with the potential value of a new treatment option. Accordingly, indications classified as PRO-dependent in this review were more likely than those classified as non-PRO-dependent to have PRO-related endpoints assessed as primary endpoints and, as a consequence, for the SmPC and package leaflet to include PRO-based information on treatment benefit. Nonetheless, further research should be undertaken to better understand the optimal positioning of PRO and non-PRO nonprimary endpoints in the statistical hierarchy to increase the likelihood that patient-reported treatment benefit is included in the SmPC and/or package leaflet.

The EMA recommends the use of a hierarchical testing strategy to assess HRQOL in confirmatory studies; the most important (efficacy) endpoints should be prioritized and HRQOL should be assessed if results on endpoints higher in the hierarchy are significant (European Medicines Agency, 2005). This may in part explain the discrepancy in the proportion of symptom-related statements compared with HRQOL-related statements we found in the SmPC and package leaflets. It may also account for disease-specific PRO measures being more common than generic measures; they have been selected or developed purposely to assess unique aspects of a disease that are considered most important to the patient population.

During the review of EPAR documents, the EQ-5D was consistently described as a measure of HRQOL when either the descriptive system or VAS were used in confirmatory studies. The EQ-5D was first developed as a generic measure that could be used as a tool to facilitate comparison of healthcare outcomes and inform decisions on resource allocation(Devlin and Brooks, 2017; Williams, 2005). It has since been widely used in submissions to health technology assessment and regulatory agencies, as well as in the wider scientific literature (Shaw et al., 2023). However, limitations have been identified in its use in clinical outcome assessment and, more specifically, for evaluating HRQOL (Gnanasakthy and DeMuro, 2024). The EQ-5D dimensional system measures HRQOL across only a limited number of domains and may not capture the full spectrum of the concept as defined by the EMA (European Medicines Agency, 2005). In their review, Shaw and colleagues (Shaw et al., 2023) found that EQ-5D data were accepted as providing evidence of clinical benefit only in a minority of health technology appraisals reviewed. Therefore, the inclusion of EQ-5D in confirmatory trials should be limited to the purpose of performing cost-utility analyses. The use of more detailed, and ideally disease-specific, HRQOL measures may provide stronger evidence to support inclusion of HRQOL data in the SmPC and package leaflet.

Findings from this review suggest that describing the impact of treatment on HRQOL in documents shared with stakeholders, such as the SmPC and package leaflet, continues to be endorsed by the EMA. They also align with previous analyses indicating that regulatory approval of HRQOL data for product value messaging is more common in submissions to the EMA than to the FDA (DeMuro et al., 2013; Marquis et al., 2011). Updated reviews with direct comparison of submissions to the FDA and EMA are needed to ascertain possible discrepancies in the evaluation and approval of PRO data by these regulatory agencies.

It is important to note that this review examined only information made publicly available by the EMA, which does not necessarily reflect all PRO data the manufacturer presents in the MAA. Further, PRO usage in extension or supportive studies was not examined. The review focused on PRO data relating to confirmatory studies, which are central to regulatory decision making on the contents of the SmPC and package leaflet. By reviewing the PAR, and the SmPC and package leaflet separately, this study is able to quantify the extent to which PRO data reviewed by the EMA are included in key documents used to communicate the value of treatment to stakeholders. The review extends previous research on PRO usage in oncology medicines approved by the EMA. Findings also reinforce the importance for the EMA and manufacturers to work collaboratively to standardize how PROs are presented in MAAs and evaluated by the EMA.

## Conclusion

Despite the positive trend in the inclusion of PROs in clinical trials, their inclusion in the SmPC and/or package leaflet remains relatively low. These documents are often the key source of information for treatment decision-making by healthcare professionals, patients, carers, and other stakeholders who do not normally access information about a medicine from the EMA website. Therefore, the inclusion of the patient experience of disease and treatment in the SmPC and package leaflet is imperative.

Findings from this review suggest that the EMA is open to a wide range of PROs, PRO measures, and endpoint types to support approval of statements of the patient-reported experience of treatment in the SmPC and/or package leaflet. Nonetheless, it appears that PRO-related statements in these documents are more common when the evidence is derived from confirmatory studies of RCT design, if they describe the benefit of treatment on symptoms or symptom burden, and/or if the PRO is assessed as a primary endpoint. Further clarity is needed on how the quality of PRO data are evaluated by the EMA. Analysis of PRO-related feedback from EMA reviewers that is published in the PAR will provide insight on reviewers’ decision making around inclusion and exclusion of PRO-related statements in the SmPC and package leaflet. Publication of a position paper by the EMA on key considerations for the inclusion of patient experience data in clinical trials will also enhance the robustness of the evidence generated and guide how PRO data should be reported in MAAs. This greater clarity will increase the likelihood that the patient experience of disease and treatment are accessible to prescribers and patients during treatment decision-making.

## Data Availability

Publicly available datasets were analyzed in this study. This data can be found here: European Medicines Agency (https://www.ema.europa.eu/en/medicines).
