# Supplementary material for: From clinical trials to informing clinical decision-making: a review of patient-reported outcomes in nononcology medicines approved by the European Medicines Agency (2018–2022)
Source: Front Pharmacol. 2025 Apr 11;16:1536401. doi: 10.3389/fphar.2025.1536401 (PMC12022438; doi:10.3389/fphar.2025.1536401)
Supplement: Supplementary file 1 [file Table1.docx]

**Additional File 1: European Public Assessment Reports (EPAR)**

| **Section** | **Information reported** | **Access avenue(s)** |
| --- | --- | --- |
| Overview | Public-friendly overview of all information | EMA website |
| Authorisation details | Key details about the product and marketing authorization holder | EMA website |
| Product Information (PI) | Summary of Product Characteristics (SmPC) – Properties and officially approved conditions of use of a medicine. Forms the basis of information for healthcare professionals on how to use the medicine safely and effectively. | EMA website; databases of the national medicine agencies; email alerts; some electronic prescribing systems |
|  | Package leaflet – The leaflet in every pack of medicine with information about the medicine for end-users, such as patients | EMA website; packaging of the medicine |
|  | Package labels – Information on the immediate or outer packaging of a medicine | EMA website; packaging of the medicine |
|  | List of all authorised presentations | EMA website |
|  | Pharmacotherapeutic group | EMA website |
| Assessment history | Public assessment report(s) (PAR) for the initial authorisation and any subsequent variations to the medicine – The assessment of a medicine and reasoning behind EMA’s decision on whether to recommend the medicine’s approval or rejection for marketing authorization. | EMA website |
|  | Orphan maintenance assessment report or withdrawal assessment report | EMA website |
|  | Overview of procedural steps taken before and after authorization | EMA website |

EMA = European Medicines Agency; PAR = Public Assessment Report(s); PI = Product Information; SmPC = Summary of Product Characteristics.

Source: European Medicines Agency. European Public Assessment Reports: Background and Context. 2010. <https://www.ema.europa.eu/en/medicines/what-we-publish-medicines-and-when/european-public-assessment-reports-background-and-context.> Accessed May 24, 2024

Note: The PAR is generally accessed via the EMA website, but health professionals often access the contents of the SmPC directly via the databases of the national medicine agencies, email alerts, and some electronic prescribing systems. The package leaflet is the only document containing information pertaining to the value of the medicine that is readily available to patients.
