# Supplementary material for: From clinical trials to informing clinical decision-making: a review of patient-reported outcomes in nononcology medicines approved by the European Medicines Agency (2018–2022)
Source: Front Pharmacol. 2025 Apr 11;16:1536401. doi: 10.3389/fphar.2025.1536401 (PMC12022438; doi:10.3389/fphar.2025.1536401)
Supplement: Supplementary file 2 [file Table2.docx]

**Additional File 2: List of Therapeutic Indications Included in Analysis (N = 140)**

| **Index** | **Brand name / generic name** | **Year of approval** | **Therapeutic indication as published by the EMA^a^** | **PRO data in the PAR** | **PRO-related statement in the SmPC and/or package leaflet** |
| --- | --- | --- | --- | --- | --- |
| 1 | Aimovig / erenumab | 2018 | Aimovig is indicated for prophylaxis of migraine in adults who have at least 4 migraine days per month. | Yes | Yes |
| 2 | Alpivab / peramivir | 2018 | Alpivab is indicated for the treatment of uncomplicated influenza in adults and children from the age of 2 years | Yes | Yes |
| 3 | Biktarvy / bictegravir, emtricitabine, tenofovir alafenamide | 2018 | Biktarvy is indicated for the treatment of human immunodeficiency virus-1 (HIV-1) infection in adults and paediatric patients at least 2 years of age and weighing at least 14 kg without present or past evidence of viral resistance to the integrase inhibitor class, emtricitabine or tenofovir (see Section 5.1) | No | No |
| 4 | Cablivi / caplacizumab | 2018 | Cablivi is indicated for the treatment of adults and adolescents of 12 years of age and older weighing at least 40 kg experiencing an episode of acquired thrombotic thrombocytopenic purpura (aTTP), in conjunction with plasma exchange and immunosuppression. | No | No |
| 5 | Pifeltro, Delstrigo / doravirine; doravirine, lamivudine, tenofovir disoproxil | 2018 | Delstrigo is indicated for the treatment of adults infected with HIV-1 without past or present evidence of resistance to the NNRTI class, lamivudine, or tenofovir (see Sections 4.4 and 5.1). Delstrigo is also indicated for the treatment of adolescents aged 12 years and older weighing at least 35 kg who are infected with HIV-1 without past or present evidence of resistance to the NNRTI class, lamivudine, or tenofovir and who have experienced toxicities which preclude the use of other regimens that do not contain tenofovir disoproxil (see Sections 4.4 and 5.1).  Pifeltro is indicated, in combination with other antiretroviral medicinal products, for the treatment of adults, and adolescents aged 12 years and older weighing at least 35 kg infected with HIV-1 without past or present evidence of resistance to the NNRTI class (see Sections 4.4 and 5.1) | No | No |
| 6 | Dengvaxia / dengue tetravalent vaccine | 2018 | Dengvaxia is indicated for the prevention of dengue disease caused by dengue virus serotypes 1, 2, 3 and 4 in individuals 6 to 45 years of age with test-confirmed previous dengue infection | No | No |
| 7 | Emgality / galcanezumab | 2018 | Emgality is indicated for the prophylaxis of migraine in adults who have at least 4 migraine days per month. | Yes | Yes |
| 8 | Hemlibra / emicizumab | 2018 | Hemlibra is indicated for routine prophylaxis of bleeding episodes in patients with haemophilia A (congenital factor VIII deficiency):  with factor VIII inhibitors  without factor VIII inhibitors who have:  severe disease (FVIII < 1%)  moderate disease (FVIII ≥ 1% and ≤ 5%) with severe bleeding phenotype.  Hemlibra can be used in all age groups. | Yes | Yes |
| 9 | Ilumetri / tildrakizumab | 2018 | Ilumetri is indicated for the treatment of adults with moderate to severe plaque psoriasis who are candidates for systemic therapy. | Yes | Yes |
| 10 | Jivi / damoctocog alfa pegol | 2018 | Treatment and prophylaxis of bleeding in previously treated patients ≥ 12 years of age with haemophilia A (congenital factor VIII deficiency). | Yes | No |
| 11 | Lamzede / velmanase alfa | 2018 | Enzyme replacement therapy for the treatment of non-neurological manifestations in patients with mild to moderate alpha-mannosidosis. | Yes | Yes |
| 12 | Luxturna / voretigene neparvovec | 2018 | Luxturna is indicated for the treatment of adult and paediatric patients with vision loss due to inherited retinal dystrophy caused by confirmed biallelic RPE65 mutations and who have sufficient viable retinal cells. | No | No |
| 13 | Mepsevii / vestronidase alfa | 2018 | Mepsevii is indicated for the treatment of non-neurological manifestations of Mucopolysaccharidosis VII (MPS VII; Sly syndrome). | Yes | Yes |
| 14 | Myalepta / metreleptin | 2018 | Myalepta is indicated as an adjunct to diet as a replacement therapy to treat the complications of leptin deficiency in lipodystrophy (LD) patients:  with confirmed congenital generalised LD (Berardinelli-Seip syndrome) or acquired generalised LD (Lawrence syndrome) in adults and children 2 years of age and above  with confirmed familial partial LD or acquired partial LD (Barraquer-Simons syndrome), in adults and children 12 years of age and above for whom standard treatments have failed to achieve adequate metabolic control. | Yes | Yes |
| 15 | Onpattro / patisiran | 2018 | Onpattro is indicated for the treatment of hereditary transthyretin-mediated amyloidosis (hATTR amyloidosis) in adult patients with stage 1 or stage 2 polyneuropathy | Yes | Yes |
| 16 | Rxulti / brexpiprazole | 2018 | RXULTI is indicated for the treatment of schizophrenia in adult patients | No | No |
| 17 | Steglatro, Segluromet, Steglujan / ertugliflozin; ertugliflozin and metformin hydrochloride; Ertugliflozin and sitagliptin | 2018 | Segluromet is indicated in adults for the treatment of type 2 diabetes mellitus as an adjunct to diet and exercise:  in patients insufficiently controlled on their maximally tolerated dose of metformin alone  in combination with other medicinal products for the treatment of diabetes in patients insufficiently controlled with metformin and these products  in patients already being treated with the combination of ertugliflozin and metformin as separate tablets.  Steglatro is indicated for the treatment of adults with insufficiently controlled type 2 diabetes mellitus as an adjunct to diet and exercise:  as monotherapy when metformin is considered inappropriate due to intolerance or contraindications.  in addition to other medicinal products for the treatment of diabetes  Steglujan is indicated in adults aged 18 years and older with type 2 diabetes mellitus as an adjunct to  diet and exercise:  to improve glycaemic control when metformin and/or a sulphonylurea (SU) and one of the monocomponents of Steglujan do not provide adequate glycaemic control.  in patients already being treated with the combination of ertugliflozin and sitagliptin as separate tablets. | No | No |
| 18 | Shingrix / herpes zoster vaccine | 2018 | Shingrix is indicated for prevention of herpes zoster (HZ) and post-herpetic neuralgia (PHN), in:  adults 50 years of age or older;  adults 18 years of age or older at increased risk of HZ.  The use of Shingrix should be in accordance with official recommendations. | Yes | Yes |
| 19 | Symkevi / tezacaftor, Ivacaftor | 2018 | Symkevi is indicated in a combination regimen with ivacaftor tablets for the treatment of patients with cystic fibrosis (CF) aged 6 years and older who are homozygous for the *F508del* mutation or who are heterozygous for the *F508del* mutation and have one of the following mutations in the cystic fibrosis transmembrane conductance regulator (*CFTR*) gene: *P67L, R117C, L206W, R352Q, A455E, D579G, 711+3A→G, S945L, S977F, R1070W, D1152H, 2789+5G→A, 3272-26A→G, and 3849+10kbC→T*. | Yes | Yes |
| 20 | Takhzyro / lanadelumab | 2018 | TAKHZYRO is indicated for routine prevention of recurrent attacks of hereditary angioedema (HAE) in patients aged 12 years and older. | Yes | Yes |
| 21 | Tegsedi / inotersen | 2018 | Tegsedi is indicated for the treatment of stage 1 or stage 2 polyneuropathy in adult patients with hereditary transthyretin amyloidosis (hATTR). | Yes | Yes |
| 22 | Vaborem / meropenem, vaborbactam | 2018 | Vaborem is indicated for the treatment of the following infections in adults (see Sections 4.4 and 5.1):  Complicated urinary tract infection (cUTI), including pyelonephritis  Complicated intra-abdominal infection (cIAI)  Hospital-acquired pneumonia (HAP), including ventilator associated pneumonia (VAP).  Treatment of patients with bacteraemia that occurs in association with, or is suspected to be associated with, any of the infections listed above.  Vaborem is also indicated for the treatment of infections due to aerobic Gram-negative organisms in adults with limited treatment options (see Sections 4.2, 4.4 and 5.1). | No | No |
| 23 | Xerava / ervacycline | 2018 | Xerava is indicated for the treatment of complicated intra-abdominal infections (cIAI) in adults | No | No |
| 24 | Mulpleo / lusutrombopag | 2018 | Mulpleo is indicated for the treatment of severe thrombocytopenia in adult patients with chronic liver disease undergoing invasive procedures (see Section 5.1) | No | No |
| 25 | Rizmoic / naldemedine | 2018 | Rizmoic is indicated for the treatment of opioid-induced constipation (OIC) in adult patients who have previously been treated with a laxative. | Yes | Yes |
| 26 | Besremi / ropeginterferon alfa-2b | 2018 | Besremi is indicated as monotherapy in adults for the treatment of polycythaemia vera without symptomatic splenomegaly. | Yes | No |
| 27 | Zynteglo / autologous CD34^+^ cells encoding β^A-T87Q^-globin gene | 2019 | Zynteglo is indicated for the treatment of patients 12 years and older with transfusion-dependent β-thalassaemia (TDT) who do not have a β^0^/β^0^ genotype, for whom haematopoietic stem cell (HSC) transplantation is appropriate but a human leukocyte antigen (HLA)-matched related HSC donor is not available (see Sections 4.4 and 5.1). | Yes | No |
| 28 | Skyrizi / risankizumab | 2019 | Skyrizi is indicated for the treatment of moderate to severe plaque psoriasis in adults who are candidates for systemic therapy. | Yes | Yes |
| 29 | Ondexxya / andexanet alfa | 2019 | For adult patients treated with a direct factor Xa (FXa) inhibitor (apixaban or rivaroxaban) when reversal of anticoagulation is needed due to life-threatening or uncontrolled bleeding. | No | No |
| 30 | Waylivra / volanesorsen | 2019 | Waylivra is indicated as an adjunct to diet in adult patients with genetically confirmed familial chylomicronemia syndrome (FCS) and at high risk for pancreatitis, in whom response to diet and triglyceride lowering therapy has been inadequate. | Yes | No |
| 31 | Ultomiris / ravulizumab | 2019 | Ultomiris is indicated in the treatment of adult and paediatric patients with a body weight of 10 kg or above with PNH: in patients with haemolysis with clinical symptom(s) indicative of high disease activity.  in patients who are clinically stable after having been treated with eculizumab for at least the past 6 months. | Yes | Yes |
| 32 | Palynziq/pegvaliase | 2019 | Palynziq is indicated for the treatment of patients with phenylketonuria (PKU) aged 16 years and older who have inadequate blood phenylalanine control (blood phenylalanine levels greater than 600 micromol/l) despite prior management with available treatment options. | Yes | Yes |
| 33 | Zynquista / sotagliflozin | 2019 | Zynquista is indicated as an adjunct to insulin therapy to improve glycaemic control in adults with type 1 diabetes mellitus with a Body Mass Index (BMI) ≥ 27 kg/m2, who have failed to achieve adequate glycaemic control despite optimal insulin therapy. | Yes | Yes |
| 34 | Ajovy / fremanezumab | 2019 | AJOVY is indicated for prophylaxis of migraine in adults who have at least 4 migraine days per month. | Yes | Yes |
| 35 | Esperoct / turoctocog alfa pegol | 2019 | Treatment and prophylaxis of bleeding in patients 12 years and above with haemophilia A (congenital factor VIII deficiency). | No | No |
| 36 | Trogarzo / ibalizumab | 2019 | Trogarzo, in combination with other antiretroviral(s), is indicated for the treatment of adults infected with multidrug resistant HIV-1 infection for whom it is otherwise not possible to construct a suppressive antiviral regimen | No | No |
| 37 | Giapreza / angiotensin II | 2019 | GIAPREZA is indicated for the treatment of refractory hypotension in adults with septic or other distributive shock who remain hypotensive despite adequate volume restitution and application of catecholamines and other available vasopressor therapies | No | No |
| 38 | Rhokiinsa / netarsudil | 2019 | Rhokiinsa is indicated for the reduction of elevated intraocular pressure (IOP) in adult patients with primary open-angle glaucoma or ocular hypertension. | No | No |
| 39 | Ervebo / Ebola Zaire vaccine | 2019 | Ervebo is indicated for active immunization of individuals 18 years of age or older to protect against Ebola Virus Disease (EVD) caused by Zaire Ebola virus | No | No |
| 40 | Rinvoq / upadacitinib | 2019 | RINVOQ is indicated for the treatment of moderate to severe active rheumatoid arthritis in adult patients who have responded inadequately to, or who are intolerant to one or more disease-modifying anti-rheumatic drugs (DMARDs). RINVOQ may be used as monotherapy or in combination with methotrexate. | Yes | Yes |
| 41 | Quofenix / delafloxacin | 2019 | Quofenix is indicated for the treatment of the following infections in adults:  acute bacterial skin and skin structure infections (ABSSSI)  community-acquired pneumonia (CAP)  when it is considered inappropriate to use other antibacterial agents that are commonly recommended  for the initial treatment of these infections (see Sections 4.4 and 5.1). | Yes | No |
| 42 | Doptelet / avatrombopag | 2019 | Doptelet is indicated for the treatment of severe thrombocytopenia in adult patients with chronic liver disease who are scheduled to undergo an invasive procedure.  Doptelet is indicated for the treatment of primary chronic immune thrombocytopenia (ITP) in adult patients who are refractory to other treatments (e.g., corticosteroids, immunoglobulins). | Yes | No |
| 43 | Tavlesse / fostamatinib | 2019 | TAVLESSE is indicated for the treatment of chronic immune thrombocytopenia (ITP) in adult patients who are refractory to other treatments | No | No |
| 44 | Recarbrio / imipenem/ cilastatin/ relebactam | 2019 | Recarbrio is indicated for:  Treatment of hospital-acquired pneumonia (HAP), including ventilator associated pneumonia (VAP), in adults (see Sections 4.4 and 5.1).  Treatment of bacteremia that occurs in association with, or is suspected to be associated with HAP or VAP, in adults.  Treatment of infections due to aerobic Gram-negative organisms in adults with limited treatment options (see Sections 4.2, 4.4, and 5.1). | No | No |
| 45 | Mayzent / siponimod | 2019 | Mayzent is indicated for the treatment of adult patients with secondary progressive multiple sclerosis (SPMS) with active disease evidenced by relapses or imaging features of inflammatory activity (see Section 5.1). | Yes | No |
| 46 | Isturisa / osilodrostat | 2019 | Isturisa is indicated for the treatment of endogenous Cushing’s syndrome in adults. | Yes | Yes |
| 47 | Sunosi / solriamfetol | 2019 | Sunosi is indicated to improve wakefulness and reduce excessive daytime sleepiness in adult patients with narcolepsy (with or without cataplexy) | Yes | Yes |
| 48 | Sunosi / solriamfetol | 2019 | Sunosi is indicated to improve wakefulness and reduce excessive daytime sleepiness (EDS) in adult patients with obstructive sleep apnoea (OSA) whose EDS has not been satisfactorily treated by primary OSA therapy, such as continuous positive airway pressure (CPAP). | Yes | Yes |
| 49 | Evenity / romosozumab | 2019 | EVENITY is indicated in treatment of severe osteoporosis in postmenopausal women at high risk of fracture | Yes | No |
| 50 | Beovu / brolucizumab | 2019 | Beovu is indicated in adults for the treatment of  neovascular (wet) age-related macular degeneration (AMD) (see Section 5.1), | Yes | Yes |
| 51 | Vocabria, Rekambys / cabotegravir, rilpivirine | 2020 | Vocabria injection is indicated, in combination with rilpivirine injection, for the treatment of Human Immunodeficiency Virus type 1 (HIV-1) infection in adults who are virologically suppressed (HIV-1 RNA <50 copies/mL) on a stable antiretroviral regimen without present or past evidence of viral resistance to, and no prior virological failure with agents of the NNRTI and INI class (see Sections 4.2, 4.4 and 5.1).  REKAMBYS is indicated, in combination with cabotegravir injection, for the treatment of human immunodeficiency virus type 1 (HIV-1) infection in adults who are virologically suppressed (HIV-1 RNA < 50 copies/mL) on a stable antiretroviral regimen without present or past evidence of viral resistance to, and no prior virological failure with, agents of the NNRTI and INI class (see Sections 4.2, 4.4 and 5.1). | Yes | No |
| 52 | Veklury / remdesivir | 2020 | Veklury is indicated for the treatment of coronavirus disease 2019 (COVID-19) in:  adults and paediatric patients (at least 4 weeks of age and weighing at least 3 kg) with pneumonia requiring supplemental oxygen (low- or high-flow oxygen or other noninvasive ventilation at start of treatment)  adults and paediatric patients (weighing at least 40 kg) who do not require supplemental oxygen and who are at increased risk of progressing to severe COVID-19 (see Section 5.1) | Yes | No |
| 53 | Libmeldy / autologous CD34^+^ cells encoding ARSA gene | 2020 | Libmeldy is indicated for the treatment of metachromatic leukodystrophy (MLD) characterized by biallelic mutations in the arylsulfatase A (ARSA) gene leading to a reduction of the ARSA enzymatic activity: in children with late infantile or early juvenile forms, without clinical manifestations of the disease | No | No |
| 54 | Kaftrio / ivacaftor / tezacaftor / elexacaftor | 2020 | Kaftrio is indicated in a combination regimen with ivacaftor for the treatment of cystic fibrosis (CF) in patients aged 6 years and older who have at least one *F508del* mutation in the cystic fibrosis transmembrane conductance regulator (*CFTR*) gene (see Section 5.1). | Yes | Yes |
| 55 | Supemtek / quadrivalent influenza vaccine (recombinant, prepared in cell culture) | 2020 | Supemtek is indicated for active immunization for the prevention of influenza disease in adults. | No | No |
| 56 | Oxlumo / lumasiran | 2020 | Oxlumo is indicated for the treatment of primary hyperoxaluria type 1 (PH1) in all age groups. | Yes | No |
| 57 | Adakveo / crizanlizumab | 2020 | Adakveo is indicated for the prevention of recurrent vaso-occlusive crises (VOCs) in sickle cell disease patients aged 16 years and older. It can be given as an add-on therapy to hydroxyurea/hydroxycarbamide (HU/HC) or as monotherapy in patients for whom HU/HC is inappropriate or inadequate. | Yes | No |
| 58 | Zolgensma / onasemnogene abeparvovec | 2020 | Zolgensma is indicated for the treatment of:  patients with 5q spinal muscular atrophy (SMA) with a bi-allelic mutation in the *SMN1* gene and a clinical diagnosis of SMA Type 1, or  patients with 5q SMA with a bi-allelic mutation in the *SMN1* gene and up to 3 copies of the *SMN2* gene | No | No |
| 59 | Staquis / crisaborole | 2020 | Staquis is indicated for treatment of mild to moderate atopic dermatitis in adults and paediatric patients from 2 years of age with ≤ 40% body surface area (BSA) affected. | Yes | No |
| 60 | Hepcludex / bulevirtide | 2020 | Hepcludex is indicated for the treatment of chronic hepatitis delta virus (HDV) infection in plasma (or serum) HDV-RNA positive adult patients with compensated liver disease. | No | No |
| 61 | Jyseleca / filgotinib | 2020 | Jyseleca is indicated for the treatment of moderate to severe active rheumatoid arthritis in adult patients who have responded inadequately to, or who are intolerant to one or more disease-modifying anti-rheumatic drugs (DMARDs). Jyseleca may be used as monotherapy or in combination with methotrexate (MTX). | Yes | Yes |
| 62 | Nustendi / bempedoic acid/ezetimibe | 2020 | Nustendi is indicated in adults with primary hypercholesterolaemia (heterozygous familial and nonfamilial) or mixed dyslipidaemia, as an adjunct to diet:  in combination with a statin in patients unable to reach LDL-C goals with the maximum tolerated dose of a statin in addition to ezetimibe (see Sections 4.2, 4.3, and 4.4),  alone in patients who are either statin-intolerant or for whom a statin is contraindicated, and are unable to reach LDL-C goals with ezetimibe alone,  in patients already being treated with the combination of bempedoic acid and ezetimibe as separate tablets with or without statin. | No | No |
| 63 | Idefirix / imlifidase | 2020 | Idefirix is indicated for desensitisation treatment of highly sensitised adult kidney transplant patients with positive crossmatch against an available deceased donor. The use of Idefirix should be reserved for patients unlikely to be transplanted under the available kidney allocation system including prioritisation programmes for highly sensitised patients. | No | No |
| 64 | Nilemdo / bempedoic acid | 2020 | Nilemdo is indicated in adults with primary hypercholesterolaemia (heterozygous familial and  non-familial) or mixed dyslipidaemia, as an adjunct to diet:  in combination with a statin or statin with other lipid-lowering therapies in patients unable to reach LDL-C goals with the maximum tolerated dose of a statin (see Sections 4.2, 4.3, and 4.4) or,  alone or in combination with other lipid-lowering therapies in patients who are statin-intolerant, or for whom a statin is contraindicated. | No | No |
| 65 | Dovprela / pretomanid | 2020 | Dovprela is indicated in combination with bedaquiline and linezolid, in adults, for the treatment of pulmonary extensively drug resistant (XDR), or treatment-intolerant or nonresponsive multidrug-resistant (MDR) tuberculosis (TB) | Yes | No |
| 66 | Xenleta / lefamulin | 2020 | Xenleta is indicated for the treatment of community-acquired pneumonia (CAP) in adults when it is considered inappropriate to use antibacterial agents that are commonly recommended for the initial treatment of CAP or when these have failed | No | No |
| 67 | Givlaari / givosiran | 2020 | Givlaari is indicated for the treatment of acute hepatic porphyria (AHP) in adults and adolescents aged 12 years and older. | Yes | Yes |
| 68 | Rukobia / fostemsavir | 2020 | Rukobia, in combination with other antiretrovirals, is indicated for the treatment of adults with multidrug resistant HIV-1 infection for whom it is otherwise not possible to construct a suppressive anti-viral regimen | No | No |
| 69 | Xofluza / baloxavir marboxil | 2020 | Xofluza is indicated for the treatment of uncomplicated influenza in patients aged 1 year and above | Yes | Yes |
| 70 | Xofluza / baloxavir marboxil | 2020 | Xofluza is indicated for post-exposure prophylaxis of influenza in individuals aged 1 year and above | Yes | Yes |
| 71 | Inrebic / fedratinib | 2020 | Inrebic is indicated for the treatment of disease-related splenomegaly or symptoms in adult patients with primary myelofibrosis, post polycythaemia vera myelofibrosis or post essential thrombocythaemia myelofibrosis who are Janus Associated Kinase (JAK) inhibitor naïve or have been treated with ruxolitinib. | Yes | Yes |
| 72 | Ultomiris / ravulizumab | 2020 | Ultomiris is indicated in the treatment of patients with a body weight of 10 kg or above with atypical haemolytic uremic syndrome (aHUS) who are complement inhibitor treatment-naïve or have received eculizumab for at least 3 months and have evidence of response to eculizumab | Yes | No |
| 73 | Rinvoq / upadacitinib | 2020 | RINVOQ is indicated for the treatment of active psoriatic arthritis in adult patients who have responded inadequately to, or who are intolerant to one or more DMARDs. RINVOQ may be used as monotherapy or in combination with methotrexate. | Yes | Yes |
| 74 | Rinvoq / upadacitinib | 2020 | Axial spondyloarthritis  *Non-radiographic axial spondyloarthritis (nr-axSpA)*  RINVOQ is indicated for the treatment of active non-radiographic axial spondyloarthritis in adult patients with objective signs of inflammation as indicated by elevated C-reactive protein (CRP) and/or magnetic resonance imaging (MRI), who have responded inadequately to nonsteroidal antiinflammatory drugs (NSAIDs).  *Ankylosing spondylitis (AS, radiographic axial spondyloarthritis)*  RINVOQ is indicated for the treatment of active ankylosing spondylitis in adult patients who have responded inadequately to conventional therapy. | Yes | Yes |
| 75 | Regkirona / regdanvimab | 2021 | Regdanvimab is indicated for the treatment of adults with coronavirus disease 2019 (COVID-19) who do not require supplemental oxygen and who are at increased risk of progressing to severe COVID-19. | Yes | Yes |
| 76 | Ronapreve / casirivimab/imdevimab | 2021 | Ronapreve is indicated for:…  Treatment of COVID-19 in adults and adolescents aged 12 years and older weighing at least 40 kg who do not require supplemental oxygen and who are at increased risk of progressing to severe COVID-19. | Yes | Yes |
| 77 | Xevudy / sotrovimab | 2021 | Xevudy is indicated for the treatment of adults and adolescents (aged 12 years and over and weighing at least 40 kg) with coronavirus disease 2019 (COVID-19) who do not require oxygen supplementation and who are at increased risk of progressing to severe COVID-19 | Yes | No |
| 78 | Kerendia / finerenone | 2021 | Kerendia is indicated for the treatment of chronic kidney disease (with albuminuria) associated with type 2 diabetes in adults. | No | No |
| 79 | Vazkepa / icosapent ethyl | 2021 | Vazkepa is indicated to reduce the risk of cardiovascular events in adult statin-treated patients at high cardiovascular risk with elevated triglycerides (≥ 150 mg/dL [≥ 1.7 mmol/L]) and  established cardiovascular disease, or  diabetes, and at least one other cardiovascular risk factor. | No | No |
| 80 | Verquvo / vericiguat | 2021 | Verquvo is indicated for the treatment of symptomatic chronic heart failure in adult patients with reduced ejection fraction who are stabilised after a recent decompensation event requiring IV therapy | Yes | Yes |
| 81 | Evkeeza / evinacumab | 2021 | Evkeeza is indicated as an adjunct to diet and other low-density lipoprotein-cholesterol (LDL-C) lowering therapies for the treatment of adult and adolescent patients aged 12 years and older with homozygous familial hypercholesterolaemia (HoFH) | Yes | No |
| 82 | Imcivree / setmelanotide | 2021 | IMCIVREE is indicated for the treatment of obesity and the control of hunger associated with genetically confirmed Bardet-Biedl syndrome (BBS), loss-of-function biallelic pro-opiomelanocortin (POMC), including PCSK1, deficiency or biallelic leptin receptor (LEPR) deficiency in adults and children 6 years of age and above. | Yes | Yes |
| 83 | Drovelis, Lydisilka / esterol, drospirenone | 2021 | Drovelis: Oral contraception. The decision to prescribe Drovelis should take into consideration the individual woman’s current risk factors, particularly those for venous thromboembolism (VTE), and how the risk of VTE with Drovelis compares with other combined hormonal contraceptives (CHCs)   Lydisilka: The decision to prescribe Lydisilka should take into consideration the individual woman’s current risk factors, particularly those for venous thromboembolism (VTE), and how the risk of VTE with Lydisilka compares with other combined hormonal contraceptives (CHCs) | Yes | No |
| 84 | Ryeqo / relugolix, estradiol, norethisterone acetate | 2021 | Ryeqo is indicated for treatment of moderate to severe symptoms of uterine fibroids in adult women of reproductive age. | Yes | Yes |
| 85 | Byfavo / remimazolam | 2021 | Remimazolam is indicated in adults for procedural sedation. | Yes | Yes |
| 86 | Evrysdi / risdiplam | 2021 | Evrysdi is indicated for the treatment of 5q spinal muscular atrophy (SMA) in patients 2 months of age and older, with a clinical diagnosis of SMA Type 1, Type 2 or Type 3 or with one to four *SMN2* copies | No | No |
| 87 | Koselugo / selumetinib | 2021 | Koselugo as monotherapy is indicated for the treatment of symptomatic, inoperable plexiform neurofibromas (PN) in paediatric patients with neurofibromatosis type 1 (NF1) aged 3 years and above. | Yes | No |
| 88 | Skysona / elivaldogene autotemcel | 2021 | Skysona is indicated for the treatment of early cerebral adrenoleukodystrophy in patients less than 18 years of age, with an ABCD1 genetic mutation, and for whom a human leukocyte antigen (HLA)-matched sibling haematopoietic stem cell (HSC) donor is not available | Yes | No |
| 89 | Vyepti / eptinezumab | 2021 | VYEPTI is indicated for the prophylaxis of migraine in adults who have at least 4 migraine days per month. | Yes | Yes |
| 90 | Skytrofa / lonapegsomatropin | 2021 | Growth failure in children and adolescents aged from 3 years up to 18 years due to insufficient endogenous growth hormone secretion (growth hormone deficiency [GHD]). | No | No |
| 91 | Ngenla / somatrogon | 2021 | Ngenla is indicated for the treatment of children and adolescents from 3 years of age with growth disturbance due to insufficient secretion of growth hormone. | Yes | No |
| 92 | Sogroya / somapacitan | 2021 | Sogroya is indicated for the replacement of endogenous growth hormone (GH) in adults with growth hormone deficiency (AGHD). | Yes | No |
| 93 | Yselty / linzagolix choline | 2021 | Yselty is indicated for the treatment of moderate to severe symptoms of uterine fibroids in adult women of reproductive age. | Yes | Yes |
| 94 | Bimzelx / bimekizumab | 2021 | Bimzelx is indicated for the treatment of moderate to severe plaque psoriasis in adults who are candidates for systemic therapy | Yes | Yes |
| 95 | Cibinqo / abrocitinib | 2021 | Cibinqo is indicated for the treatment of moderate-to-severe atopic dermatitis in adults who are candidates for systemic therapy. | Yes | Yes |
| 96 | Klisyri / tirbanibulin | 2021 | Klisyri is indicated for the field treatment of non-hyperkeratotic, non-hypertrophic actinic keratosis (Olsen grade 1) of the face or scalp in adults. | No | No |
| 97 | Enspryng / satralizumab | 2021 | Enspryng is indicated as a monotherapy or in combination with immunosuppressive therapy (IST) for the treatment of neuromyelitis optica spectrum disorders (NMOSD) in adult and adolescent patients from 12 years of age who are anti-aquaporin-4 IgG (AQP4-IgG) seropositive | Yes | Yes |
| 98 | Uplizna / inebilizumab | 2021 | Uplizna is indicated as monotherapy for the treatment of adult patients with neuromyelitis optica spectrum disorders (NMOSD) who are anti-aquaporin-4 immunoglobulin G (AQP4-IgG) seropositive | No | No |
| 99 | Artesunate Amivas / artesunate | 2021 | Artesunate Amivas is indicated for the initial treatment of severe malaria in adults and children (see Sections 4.2 and 5.1). Consideration should be given to official guidance on the appropriate use of antimalarial agents. | No | No |
| 100 | Saphnelo / anifrolumab | 2021 | Saphnelo is indicated as an add-on therapy for the treatment of adult patients with moderate to severe, active autoantibody-positive systemic lupus erythematosus (SLE), despite standard therapy. | Yes | No |
| 101 | Tavneos / avacopan | 2021 | Tavneos, in combination with a rituximab or cyclophosphamide regimen, is indicated for the treatment of adult patients with severe, active granulomatosis with polyangiitis (GPA) or microscopic polyangiitis (MPA) | Yes | No |
| 102 | Voxzogo / vosoritide | 2021 | Voxzogo is indicated for the treatment of achondroplasia in patients 2 years of age and older whose epiphyses are not closed. The diagnosis of achondroplasia should be confirmed by appropriate genetic testing. | Yes | No |
| 103 | Evrenzo / roxadustat | 2021 | Evrenzo is indicated for treatment of adult patients with symptomatic anaemia associated with chronic kidney disease (CKD). | Yes | Yes |
| 104 | Oxbryta / voxelotor | 2021 | Oxbryta is indicated for the treatment of haemolytic anaemia due to sickle cell disease (SCD) in adults and paediatric patients 12 years of age and older as monotherapy or in combination with hydroxycarbamide. | Yes | No |
| 105 | Skyrizi / risankizumab | 2021 | Skyrizi, alone or in combination with methotrexate (MTX), is indicated for the treatment of active psoriatic arthritis in adults who have had an inadequate response or who have been intolerant to one or more disease-modifying antirheumatic drugs (DMARDs). | Yes | Yes |
| 106 | Rinvoq / upadacitinib | 2021 | RINVOQ is indicated for the treatment of moderate to severe atopic dermatitis in adults and adolescents 12 years and older who are candidates for systemic therapy. | Yes | Yes |
| 107 | Jyseleca / filgotinib | 2021 | Jyseleca is indicated for the treatment of adult patients with moderately to severely active ulcerative colitis who have had an inadequate response with, lost response to, or were intolerant to either conventional therapy or a biologic agent. | Yes | Yes |
| 108 | Hemgenix / etranacogene dezaparvovec | 2022 | Hemgenix is indicated for the treatment of severe and moderately severe Haemophilia B (congenital Factor IX deficiency) in adult patients without a history of Factor IX inhibitors. | Yes | No |
| 109 | Roctavian / valoctocogene roxaparvovec | 2022 | ROCTAVIAN is indicated for the treatment of severe haemophilia A (congenital factor VIII deficiency) in adult patients without a history of factor VIII inhibitors and without detectable antibodies to adeno-associated virus serotype 5 (AAV5). | Yes | No |
| 110 | Nulibry / fosdenopterin | 2022 | NULIBRY is indicated for the treatment of patients with molybdenum cofactor deficiency (MoCD) Type A. | No | No |
| 111 | Pyrukynd / mitapivat | 2022 | Pyrukynd is indicated for the treatment of pyruvate kinase deficiency (PK deficiency) in adult patients | Yes | No |
| 112 | Xenpozyme / olipudase alfa | 2022 | Xenpozyme is indicated as an enzyme replacement therapy for the treatment of non-Central Nervous System (CNS) manifestations of Acid Sphingomyelinase Deficiency (ASMD) in paediatric and adult patients with type A/B or type B. | Yes | Yes |
| 113 | Amvuttra / vutrisiran | 2022 | Amvuttra is indicated for the treatment of hereditary transthyretin-mediated amyloidosis (hATTR amyloidosis) in adult patients with stage 1 or stage 2 polyneuropathy. | Yes | Yes |
| 114 | Quviviq / daridorexant | 2022 | QUVIVIQ is indicated for the treatment of adult patients with insomnia characterised by symptoms present for at least 3 months and considerable impact on daytime functioning. | Yes | Yes |
| 115 | Rayvow / lasmiditan | 2022 | RAYVOW is indicated for the acute treatment of the headache phase of migraine attacks, with or without aura in adults. | Yes | Yes |
| 116 | Upstaza / eladocagene exuparvovec | 2022 | Upstaza is indicated for the treatment of patients aged 18 months and older with a clinical, molecular, and genetically confirmed diagnosis of aromatic L-amino acid decarboxylase (AADC) deficiency with a severe phenotype | No | No |
| 117 | Vydura / rimegepant | 2022 | VYDURA is indicated for the  Acute treatment of migraine with or without aura in adults; | Yes | Yes |
| 118 | Vydura / rimegepant | 2022 | VYDURA is indicated for the…  Preventive treatment of episodic migraine in adults who have at least 4 migraine attacks per month | Yes | Yes |
| 119 | Vyvgart / efgartigimod alfa | 2022 | Vyvgart is indicated as an add-on to standard therapy for the treatment of adult patients with generalised Myasthenia Gravis (gMG) who are anti-acetylcholine receptor (AChR) antibody positive. | Yes | Yes |
| 120 | Evusheld / tixagevimab/cilgavimab | 2022 | EVUSHELD is indicated for the pre-exposure prophylaxis of COVID-19 in adults and adolescents aged 12 years and older weighing at least 40 kg | Yes | No |
| 121 | Evusheld / tixagevimab/cilgavimab | 2022 | EVUSHELD is indicated for the treatment of adults and adolescents (aged 12 years and older weighing at least 40 kg) with COVID-19, who do not require supplemental oxygen and who are at increased risk of progressing to severe COVID-19 | Yes | No |
| 122 | Paxlovid / nirmatrelvir/ritonavir | 2022 | Paxlovid is indicated for the treatment of coronavirus disease 2019 (COVID-19) in adults who do not require supplemental oxygen and who are at increased risk for progressing to severe COVID-19 | Yes | No |
| 123 | Beyfortus / nirsevimab | 2022 | Beyfortus is indicated for the prevention of Respiratory Syncytial Virus (RSV) lower respiratory tract disease in neonates and infants during their first RSV season. | No | No |
| 124 | Livtencity / maribavir | 2022 | LIVTENCITY is indicated for the treatment of cytomegalovirus (CMV) infection and/or disease that are refractory (with or without resistance) to one or more prior therapies, including ganciclovir, valganciclovir, cidofovir or foscarnet in adult patients who have undergone a haematopoietic stem cell transplant (HSCT) or solid organ transplant (SOT). | Yes | Yes |
| 125 | Sunlenca / lenacapavir | 2022 | Sunlenca injection, in combination with other antiretroviral(s), is indicated for the treatment of adults with multidrug-resistant HIV-1 infection for whom it is otherwise not possible to construct a suppressive anti-viral regimen (see Sections 4.2 and 5.1). | No | No |
| 126 | Eladynos / abaloparatide | 2022 | Treatment of osteoporosis in postmenopausal women at increased risk of fracture | No | No |
| 127 | Mounjaro / tirzepatide | 2022 | Mounjaro is indicated for the treatment of adults with insufficiently controlled type 2 diabetes mellitus as an adjunct to diet and exercise as monotherapy when metformin is considered inappropriate due to intolerance or contraindications in addition to other medicinal products for the treatment of diabetes. | Yes | No |
| 128 | Enjaymo / sutimlimab | 2022 | Enjaymo is indicated for the treatment of haemolytic anaemia in adult patients with cold agglutinin disease (CAD). | Yes | Yes |
| 129 | Kapruvia / difelikefalin | 2022 | Kapruvia is indicated for the treatment of moderate-to-severe pruritus associated with chronic kidney disease in adult patients on haemodialysis | Yes | Yes |
| 130 | Lupkynis / voclosporin | 2022 | Lupkynis is indicated in combination with mycophenolate mofetil for the treatment of adult patients with active class III, IV or V (including mixed class III/V and IV/V) lupus nephritis (LN). | Yes | No |
| 131 | Vabysmo / faribimab | 2022 | Vabysmo is indicated for the treatment of adult patients with: neovascular (wet) age-related macular degeneration (nAMD), | Yes | Yes |
| 132 | Vabysmo / faribimab | 2022 | Vabysmo is indicated for the treatment of adult patients with:…  visual impairment due to diabetic macular oedema (DME). | Yes | Yes |
| 133 | Qdenga / dengue tetravalent vaccine (live, attenuated) | 2022 | Qdenga is indicated for the prevention of dengue disease in individuals from 4 years of age. | No | No |
| 134 | Tezspire / tezepelumab | 2022 | Tezspire is indicated as an add-on maintenance treatment in adults and adolescents 12 years and older with severe asthma who are inadequately controlled despite high dose inhaled corticosteroids plus another medicinal product for maintenance treatment. | Yes | Yes |
| 135 | Spevigo / spesolimab | 2022 | Spevigo is indicated for the treatment of flares in adult patients with generalised pustular psoriasis (GPP) as monotherapy. | Yes | Yes |
| 136 | Zokinvy / lonafarnib | 2022 | Zokinvy is indicated for the treatment of patients 12 months of age and older with a genetically confirmed diagnosis of Hutchinson-Gilford progeria syndrome or a processing-deficient progeroid laminopathy associated with either a heterozygous *LMNA* mutation with progerin-like protein accumulation or a homozygous or compound heterozygous *ZMPSTE24* mutation. | No | No |
| 137 | Skyrizi / risankizumab | 2022 | Skyrizi is indicated for the treatment of adult patients with moderately to severely active Crohn’s disease who have had an inadequate response to, lost response to, or were intolerant to conventional therapy or a biologic therapy. | Yes | Yes |
| 138 | Ultomiris / ravulizumab | 2022 | Ultomiris is indicated as an add-on to standard therapy for the treatment of adult patients with gMG who are anti-acetylcholine receptor (AChR) antibody-positive. | Yes | Yes |
| 139 | Rinvoq / upadacitinib | 2022 | RINVOQ is indicated for the treatment of adult patients with moderately to severely active ulcerative colitis who have had an inadequate response, lost response or were intolerant to either conventional therapy or a biologic agent. | Yes | Yes |
| 140 | Beovu / brolucizumab | 2022 | Beovu is indicated in adults for the treatment of…  visual impairment due to diabetic macular oedema (DME) (see Section 5.1). | Yes | No |

ABSSSI = acute bacterial skin and skin structure infection; AChR = anti-acetylcholine receptor; AGHD = adults with growth hormone deficiency; AHP = acute hepatic porphyria; aHUS = atypical hemolytic uremic syndrome; AMD = age-related macular degeneration; AQP4-IgG = anti-aquaporin-4 IgG; ARSA = arylsulfatase A; AS = ankylosing spondylitis; ASMD = acid sphingomyelinase deficiency; aTTP = acquired thrombotic thrombocytopenic purpura; BBS = Bardet-Biedl syndrome; BMI = body mass index; BSA = body surface area; CAD = cold agglutinin disease; CAP = community-acquired pneumonia; CF = cystic fibrosis; CFTR = cystic fibrosis transmembrane conductance regulator; CHC = combined hormonal contraceptive; cIAI = complicated intra-abdominal infection; CKD = chronic kidney disease; CMV = cytomegalovirus; CNS = central nervous system; COVID-19 = coronavirus disease 2019; CPAP = continuous positive airway pressure; CRP = C-reactive protein; cUTI = complicated urinary tract infection; DMARD = disease-modifying antirheumatic drug; DME = diabetic macular oedema; EDS = excessive daytime sleepiness; EVD = Ebola virus disease; FCS = familial chylomicronemia syndrome; FXa = factor Xa; GH = growth hormone; GHD = growth hormone deficiency; gMG = generalized Myasthenia gravis; GPA = granulomatosis with polyangiitis; GPP = generalized pustular psoriasis; HAE = hereditary angioedema; HAP = hospital-acquired pneumonia; hATTR = hereditary transthyretin amyloidosis; HDV = hepatitis delta virus; HIV-1 = human immunodeficiency virus type 1; HLA = human leukocyte antigen; HoFH = homozygous familial hypercholesterolemia; HSC = hematopoietic stem cell; HSCT = hematopoietic stem cell transplant; HU/HC = hydroxyurea/hydroxycarbamide; HZ = herpes zoster; IOP = intraocular pressure; IST = immunosuppressive therapy; ITP = immune thrombocytopenia; IV = intravenous; JAK = Janus associated kinase; LD = lipodystrophy; LDL-C = low-density lipoprotein-cholesterol; LEPR = biallelic leptin receptor; LN = lupus nephritis; MAH = market authorization holder; MDR = multidrug-resistant; MLD = metachromatic leukodystrophy; MoCD = molybdenum cofactor deficiency; MPA = microscopic polyangiitis; MPS VII = Mucopolysaccharidosis VII; MRI = magnetic resonance imaging; MTX = methotrexate; nAMD = neovascular (wet) age-related macular degeneration; NF1 = neurofibromatosis type 1; NMOSD = neuromyelitis optica spectrum disorder; nr-axSpA = nonradiographic axial spondyloarthritis; NSAID = nonsteroidal anti-inflammatory drug; OIC = opioid-induced constipation; OSA = obstructive sleep apnea; PAR = Public Assessment Report (CHMP review of MAA); PCSK1 = proprotein convertase subtilisin/kexin Type 1; PHN = post-herpetic neuralgia; PH1 = primary hyperoxaluria type 1; PI = Product Information (contains SmPC and package leaflet); PK = pyruvate kinase; PKU = phenylketonuria; PN = plexiform neurofibroma; POMC = pro-opiomelanocortin; PRO = patient-reported outcome; RSV = Respiratory Syncytial Virus; SCD = sickle cell disease; SLE = systemic lupus erythematosus; SMA = spinal muscular atrophy; SOT = solid organ transplant; SPMS = secondary progressive multiple sclerosis; SU = sulphonylurea; TB = tuberculosis; TDT = transfusion-dependent β-thalassemia; VAP = ventilator associated pneumonia; VOC = vaso-occlusive crisis; VTE = venous thromboembolism; XDR = extensively drug resistant

^a^ All information about each indication can be found on the EMA website (<https://www.ema.europa.eu/en/homepage>), by searching for the product name.
